# Supplementary material for: All-flexible chronoepifluidic nanoplasmonic patch for label-free metabolite profiling in sweat
Source: Nat Commun. 2025 Aug 27;16:8017. doi: 10.1038/s41467-025-63510-2 (PMC12391418; doi:10.1038/s41467-025-63510-2)
Supplement: Supplementary file 1 — Supplementary Information [file 41467_2025_63510_MOESM1_ESM.pdf]

Supporting Information

All-Flexible Chronoepifluidic Nanoplasmonic Patch for Label-free Metabolite Profiling in Sweat

Jaehun Jeon[1,2], Sangyeon Lee[1], Seongok Chae[3], Joo Hoon Lee[4], Hanjin Kim[1], Eun-Sil Yu[1,2], Hamin Na[1,2], Taejoon Kang[4], Hyung-Soon Park[3], Doheon Lee[1], and Ki-Hun Jeong[1,2,*]

[1] *Department of Bio and Brain Engineering, Korea Advanced Institute of Science and Technology (KAIST), 291 Daehak-ro, Yuseong-gu, Daejeon 34141, Republic of Korea*
[2] *KAIST Institute for Health Science and Technology (KIHST), KAIST, 291 Daehak-ro, Yuseong-gu, Daejeon, Republic of Korea, 305-701*
[3] *Department of Mechanical Engineering, Korea Advanced Institute of Science and Technology (KAIST), 291 Daehak-ro, Yuseong-gu, Daejeon, 34141, Korea*
[4] *Bionanotechnology Research Center, Korea Research Institute of Bioscience and Biotechnology (KRIBB), 125 Gwahak-ro, Yuseong-gu, Daejeon, Republic of Korea, 350-701*
[5] *School of Pharmacy, Sungkyunkwan University (SKKU), 2066 Seobu-ro, Jangan-gu, Suwon 16419, Republic of Korea*

**kjeong@kaist.ac.kr*

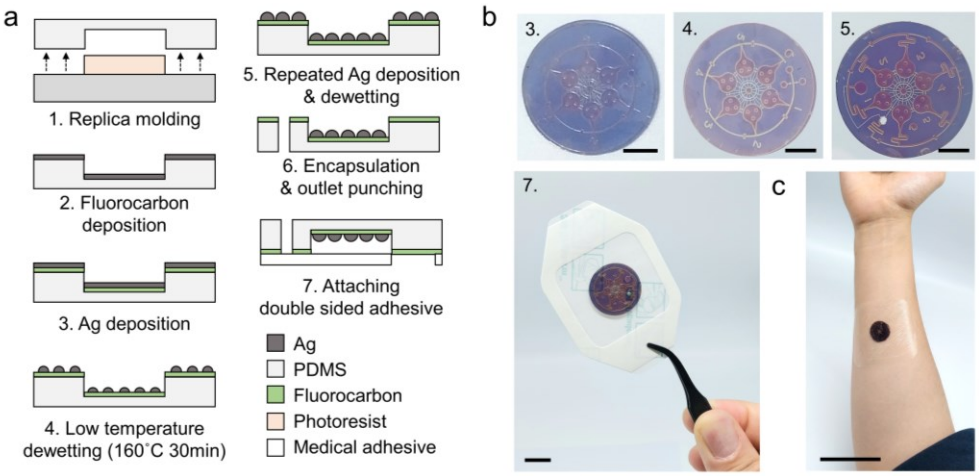

Figure S1. (**a**) Detailed micro and nanofabrication of the CEP-SERS patch. (**b**) optical images of the fabrication step 3, 4, 5 (Scale bar: 5 mm), 7 (Scale bar: 10 mm), and (**c**) an optical image of the CEP-SERS patch conformally attached to human skin (Scale bar: 60 mm).

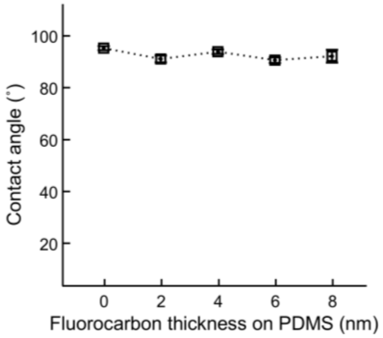

Figure S2. Measured contact angle depending on the fluorocarbon thickness on PDMS. The error bars indicate one standard deviation from the mean (n=5 technical replicates).

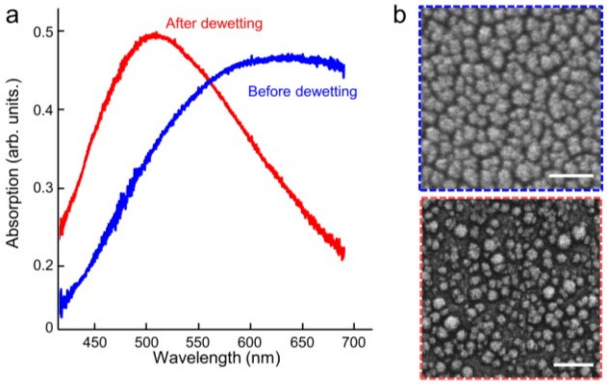

Figure S3. (**a**) Absorption spectra and (**b**) SEM images before (blue) and after (red) low-temperature solid-state dewetting of Ag thin film on fluorocarbon-coated PDMS (Scale bar: 100 nm). The Ag film and fluorocarbon thickness are 10 nm and 2 nm, respectively.

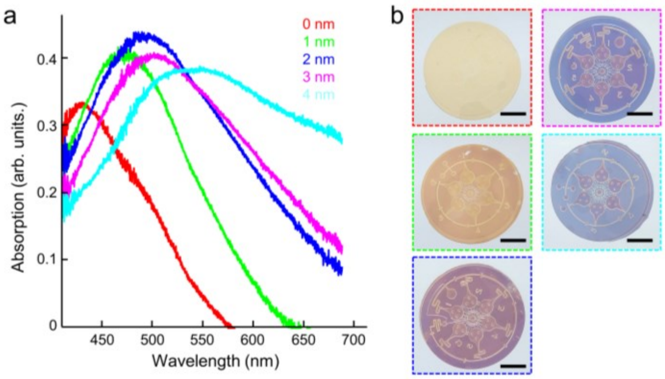

Figure S4. (**a**) Absorption spectra and (**b**) optical images of the Ag nanoislands on fluorocarbon-coated PDMS. Each color represents different fluorocarbon thicknesses. (Scale bar: 5 mm).

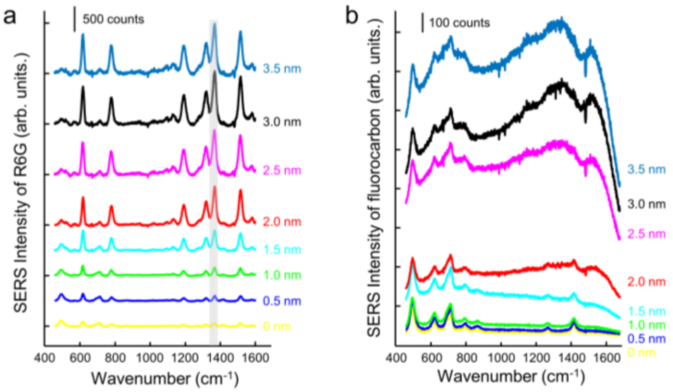

Figure S5. (**a**) SERS spectra of 10 μM R6G (**b**) SERS noise spectra of fluorocarbon depending on fluorocarbon thicknesses. The gray bars represent the wavenumber of 1365 cm^{-1}.

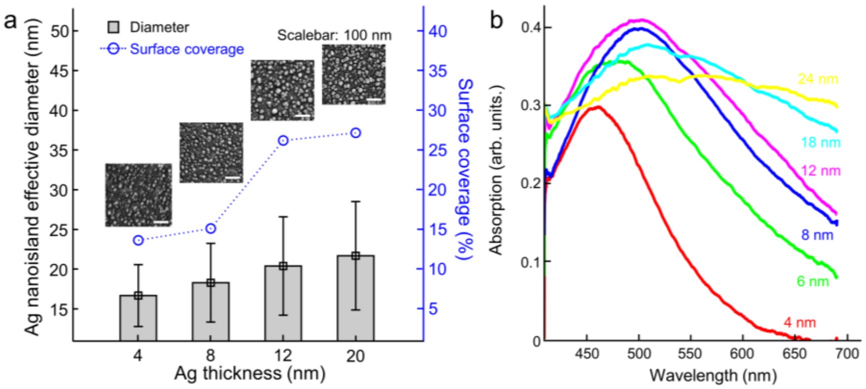

Figure S6. (**a**) Measured diameter, packing density, and (**b**) absorption spectra of Ag nanoisland on fluorocarbon-coated PDMS depending on Ag film thickness. The error bars represent one standard deviation from the mean of three different SEM images.

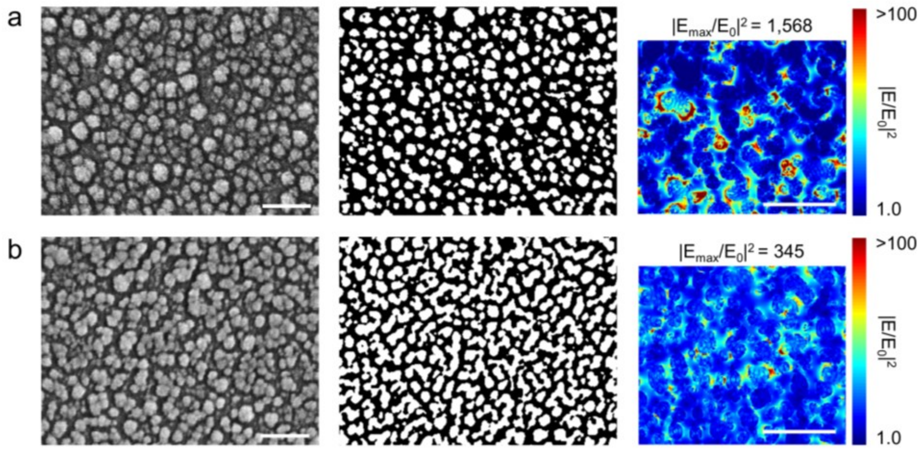

Figure S7. SEM images (left), binary images (middle), and calculated E-field enhancement (right) of Ag nanoislands on fluorocarbon-coated PDMS fabricated by (**a**) 10 nm and (**b**) 12 nm repeated dewetting (Scale bar: 100nm).

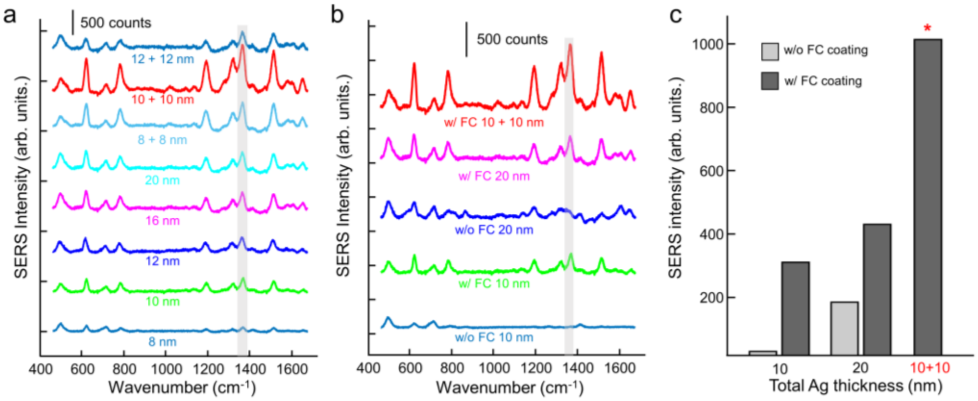

Figure S8. SERS spectra of 1 μM R6G using SERS substrate fabricated by (**a**) different Ag film thickness, dewetting repetition, and (**b**) fluorocarbon (FC) coating. The gray bars represent the wavenumber of 1365 cm^{-1}. (**c**) Comparison of SERS peak intensities from different nanofabrication conditions.

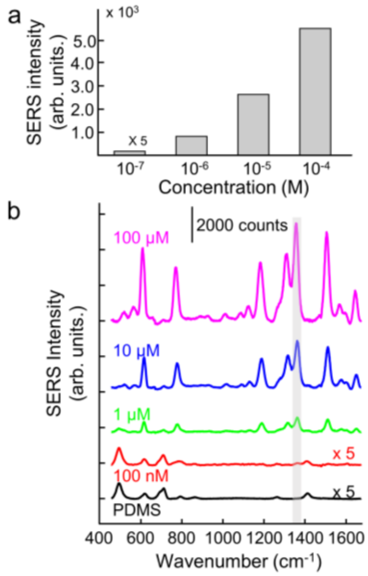

Figure S9. (**a**) SERS intensity at 1365 cm^{-1} depends on different concentrations of R6G. (**b**) SERS spectra of R6G depending on different concentrations. The gray bars represent the wavenumber of 1365 cm^{-1}.

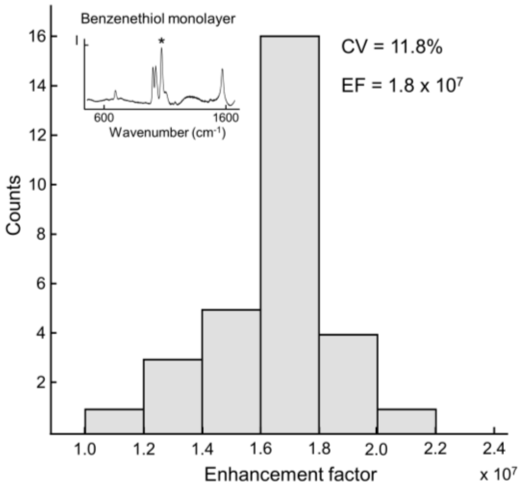

Figure S10. SERS enhancement factor and fabrication uniformity of the CEP-SERS patch measured from 30 different points.

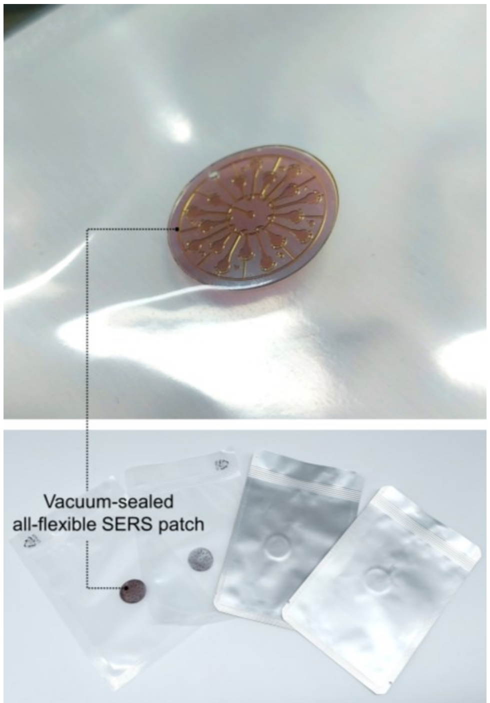

Figure S11. Optical images of vacuum-sealed CEP-SERS patch for on-body evaluation.

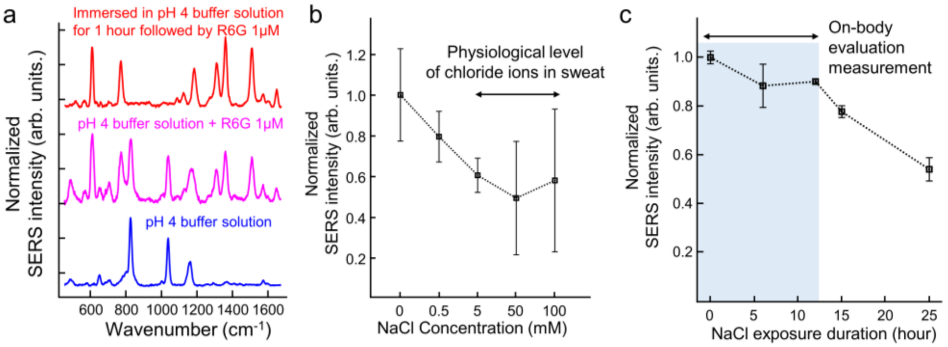

Figure S12. (**a**) Measured SERS Spectra of pH 4 buffer solution (blue), 1 μM R6G in the buffer solution (magenta), and 1 μM R6G after 1 hour immersion in the buffer solution (red). (**b**) SERS performance stability evaluated by 1 μM R6G SERS intensity measurement at 1365 cm^{-1} under different concentrations of NaCl (n=9 technical replicates). (**c**) Long-term stability of the CEP-SERS patch in 50mM NaCl solution measured by SERS intensity of 1 μM R6G (n=3 technical replicates). The error bars represent one standard deviation from the mean.

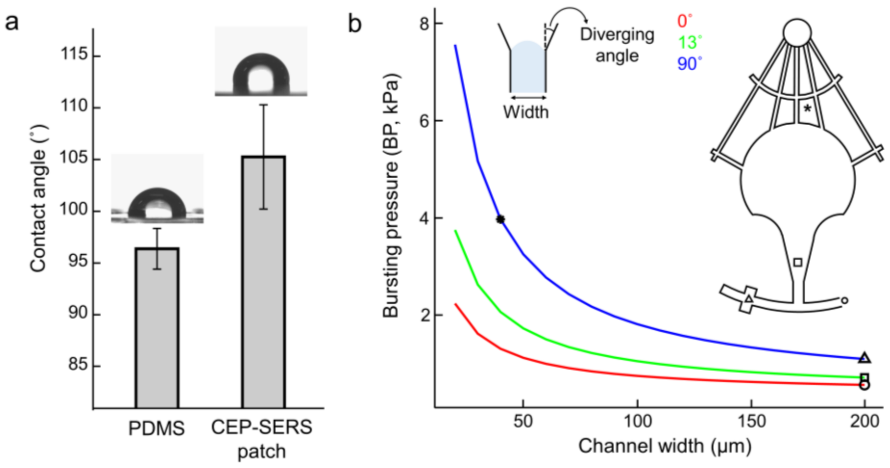

Figure S13. (**a**) The measured contact angle of PDMS and CEP-SERS patch (n=4 technical replicates). (**b**) Bursting pressure (BP) calculated by the Young-Laplace equation depending on channel widths and diverging angles. Each marker represents a specific CBV and its BP. The error bars represent one standard deviation from the mean.

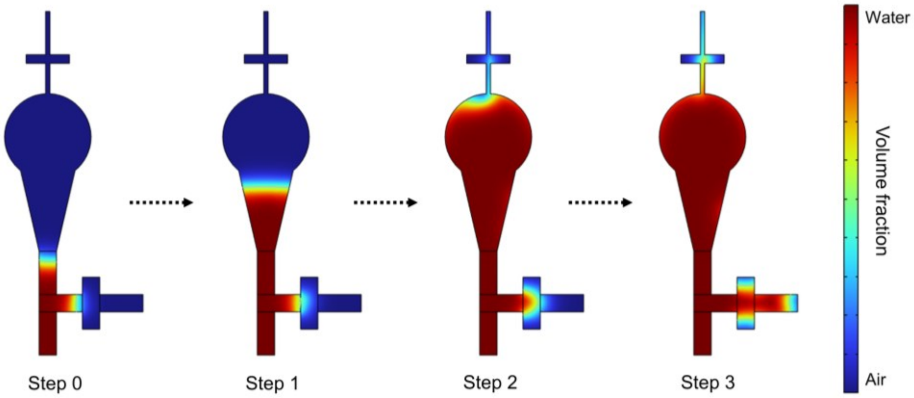

Figure S14. Two-phase fluid dynamic analysis of chrono-sampling through capillary bursting valves.

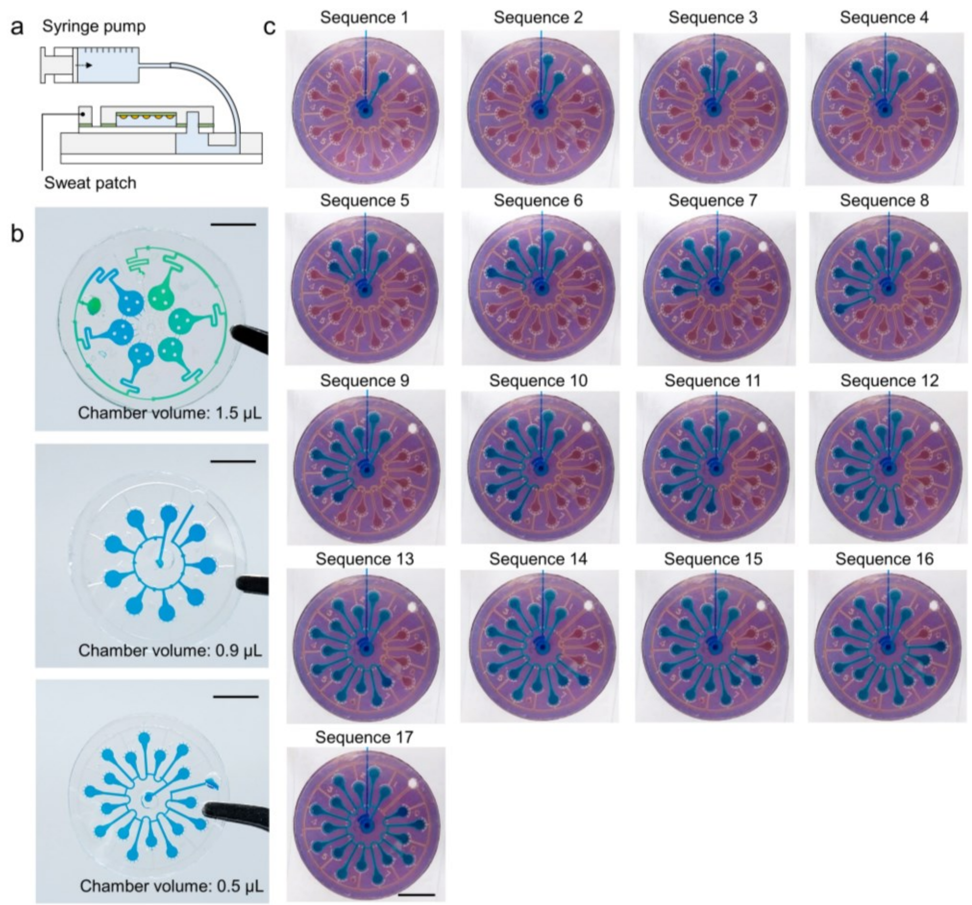

Figure S15. (**a**) Schematic illustration of sequential sample injection setup. Optical images of (**b**) microfluidic sequential sampler with different chamber volumes and (**c**) sequential sampling using the CEP-SERS patch with 0.5 μL chamber volume (Scale bar: 5 mm).

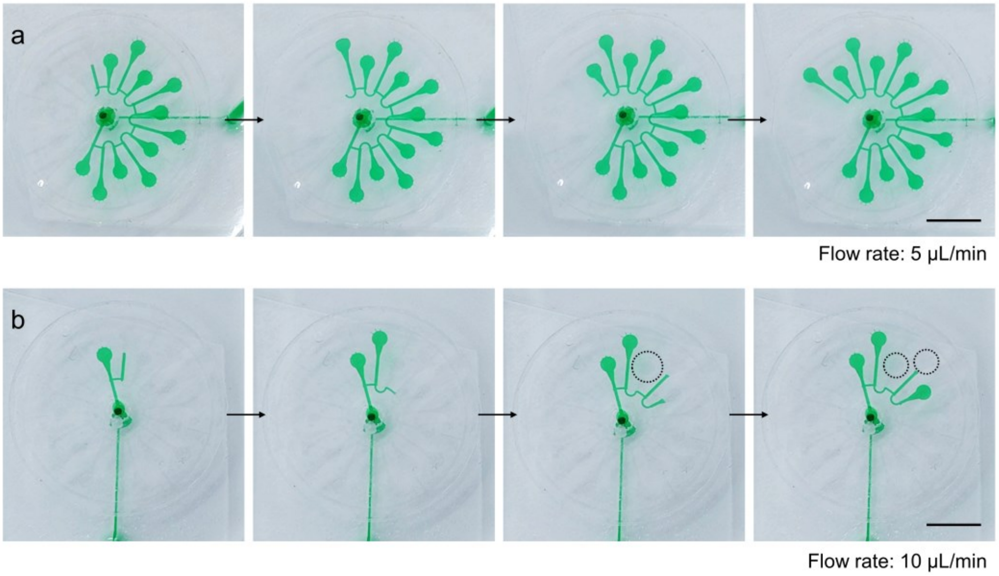

Figure S16. CBV operation under different flow rate of (**a**) 5 µL/min and (**b**) 10 µL/min. The dotted circles represent skipped chamber during sequential sampling due to unstable capillary bursting valve operation. The scale bar represents 5 mm.

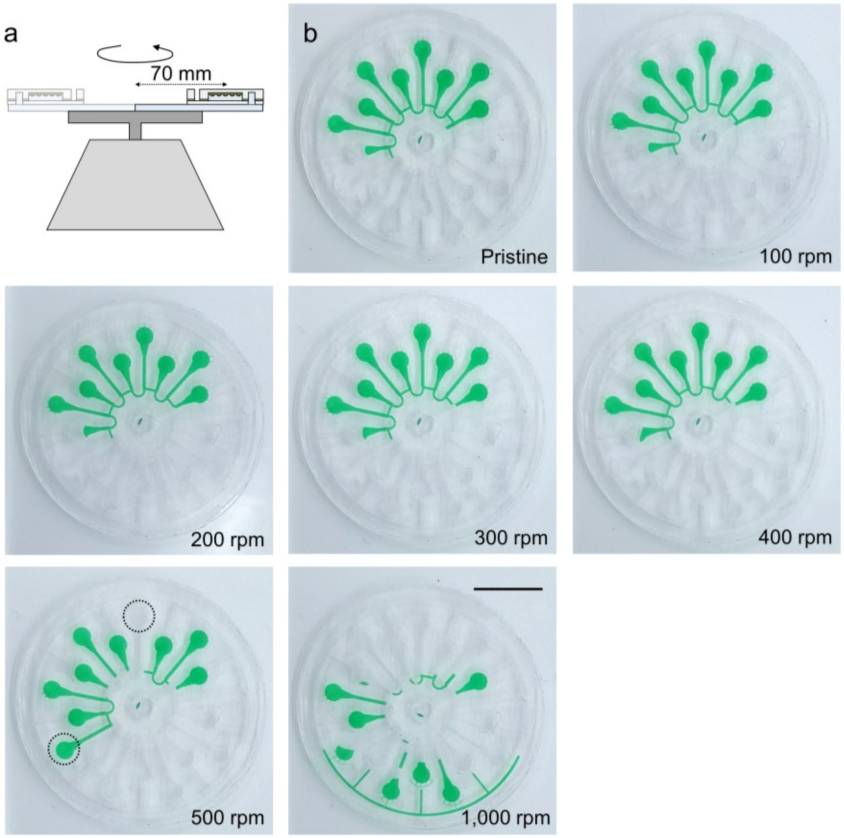

Figure S17. (**a**) Schematic illustration of test setup for acceleration stability of microfluidic sampler. (**b**) Optical images of microfluidic sampler under different rpm. The dotted circles represent the fluid shifting after rotation. The scale bar represents 5 mm.

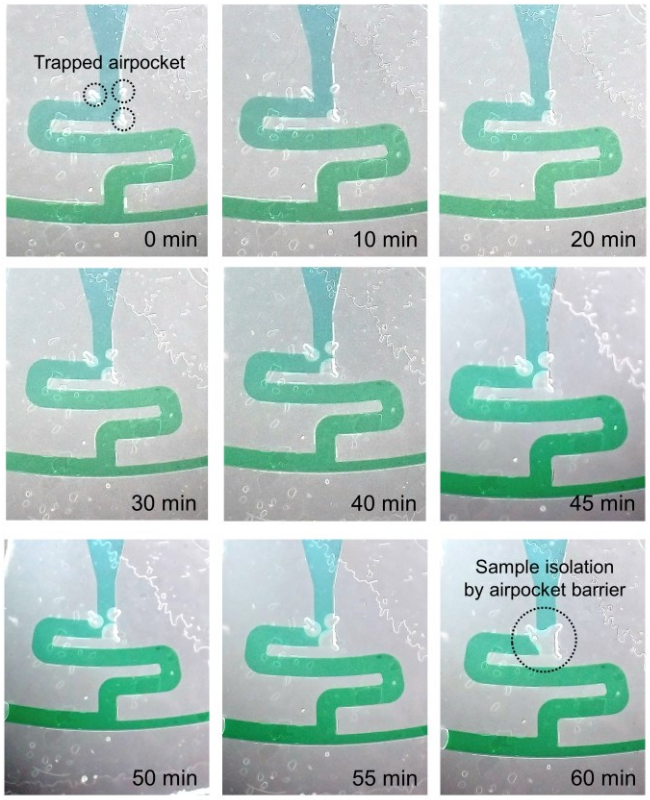

Figure S18. Time-lapse images of sample isolation driven by natural evaporation of the sample in contact with a trapped air pocket.

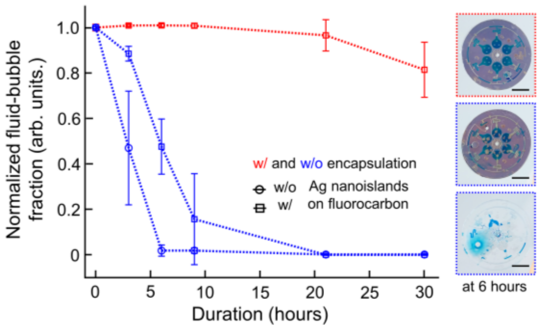

Figure S19. Normalized fluid and bubble fraction (n=6 technical replicates) depending on storage duration with and without Ag nanoisland on fluorocarbon and encapsulation (Scale bar: 5mm). Right optical images show the sample storage after 6 hours. The error bars indicate one standard deviation from the mean.

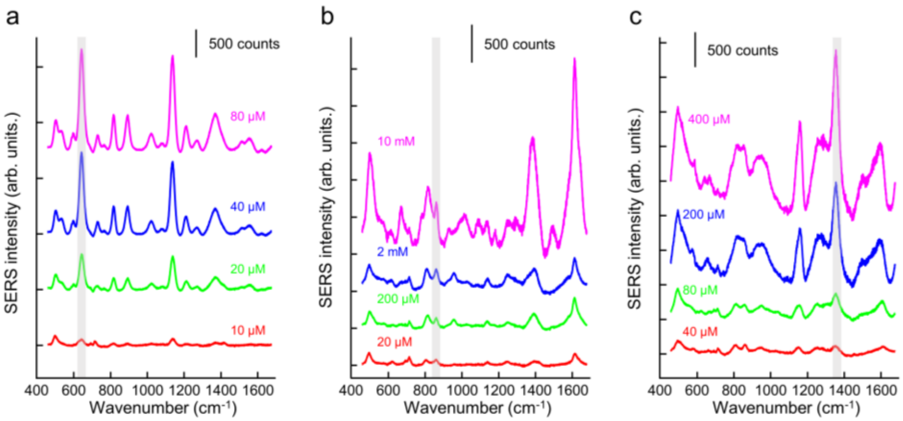

Figure S20. SERS spectra of (**a**) uric acid, (**b**) lactate, and (**c**) tyrosine depending on different concentrations. The gray bars represent the main SERS peak of each metabolite.

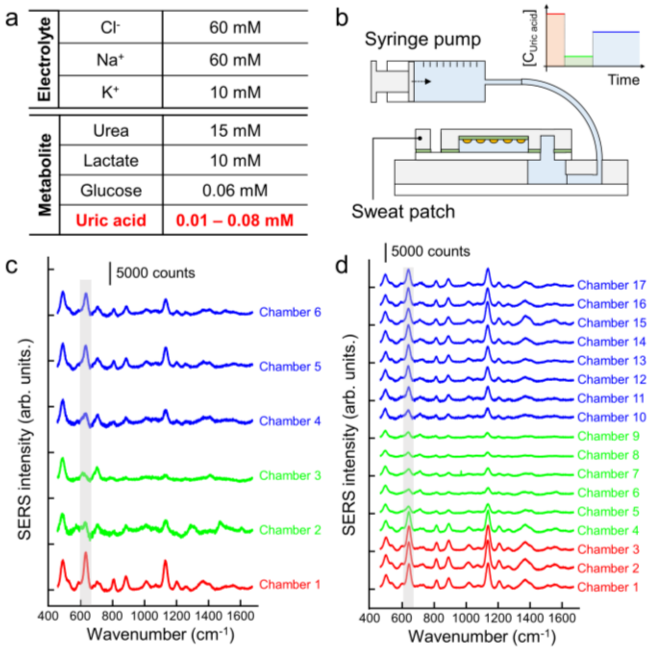

Figure S21. (**a**) Composition of the artificial sweat solution. (**b**) Schematic illustration for sequential injection of the artificial sweat solution with different uric acid concentrations. SERS spectra from chrono-sampled solution with (**c**) sparse and (**d**) dense sampling intervals.

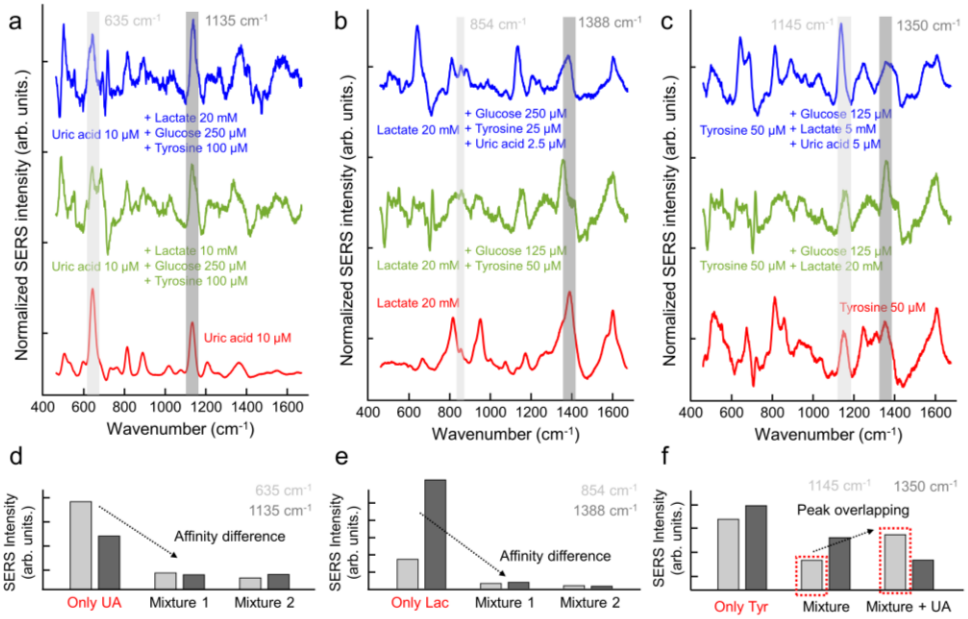

Figure S22. Examples of SERS spectra for (**a**) uric acid, (**b**) lactate, and (**c**) tyrosine in different mixture solutions. Each SERS spectrum is an average of 36 different measurements. SERS intensity of (**d**) uric acid, (**e**) lactate, and (**f**) tyrosine under varying background molecular conditions.

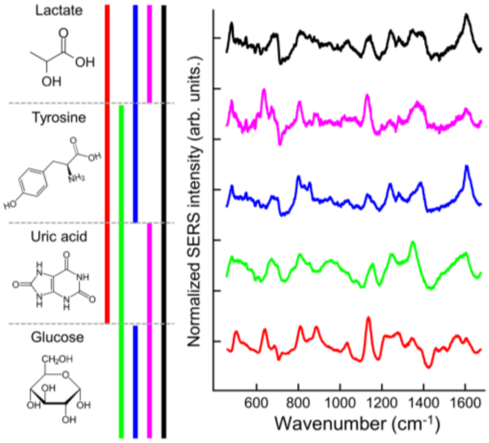

Figure S23. SERS spectra examples of mixture solutions with different combinations of variable metabolites. Each SERS spectrum is an average of 36 different measurements.

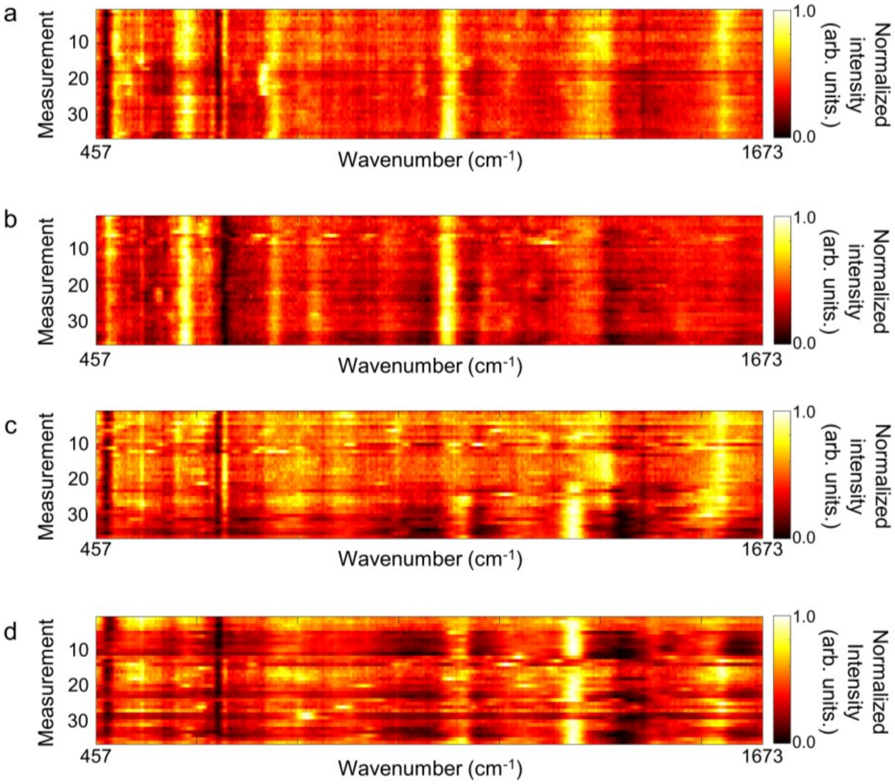

Figure S24. Examples of SERS spectra for training of metabolic quantification model. Each colormap represent specific combination of metabolites: (**a**) 5 mM lactate, 2.5 µM uric acid, 25 µM tyrosine, and 250 µM glucose (**b**) 10 mM lactate, 20 µM uric acid, 100 µM tyrosine, and 250 µM glucose**,** (**c**) 20 mM lactate, 50 µM tyrosine, and 125 µM glucose**,** (**d**) 10 mM lactate, 50 µM tyrosine, and 125 µM glucose mixed in base solution. A total of 36 SERS spectra were acquired and used for training at each concentration combination to account for signal fluctuations during measurement.

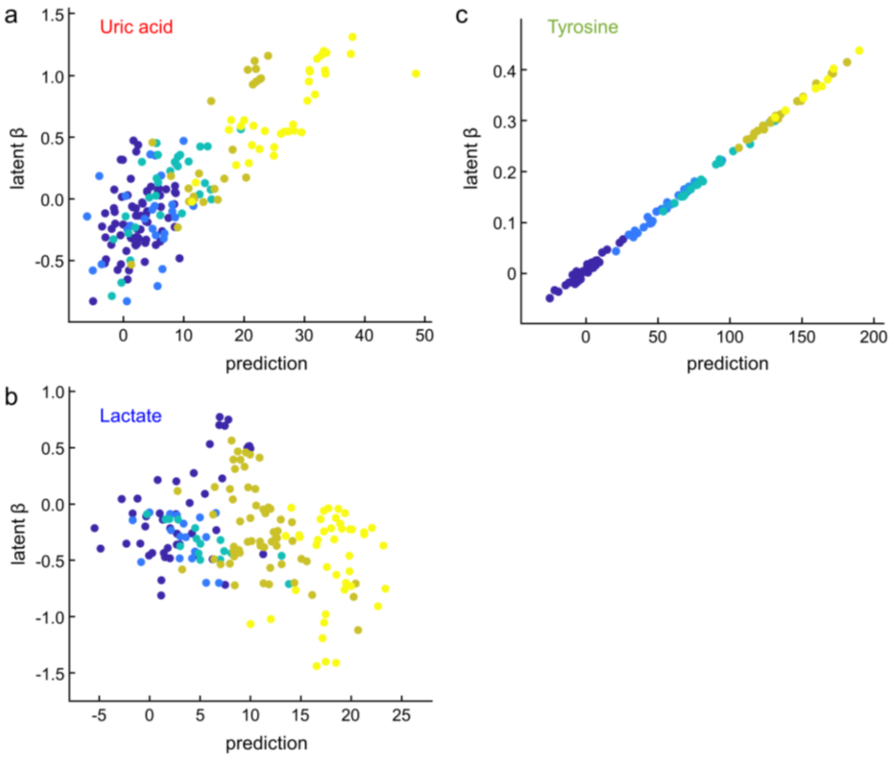

Figure S25. Two-dimensional latent scores of (**a**) uric acid, (**b**) lactate, and (**c**) tyrosine from measured SERS signals with different concentrations. Each color displays the different concentrations of the target metabolite in the mixture solution.

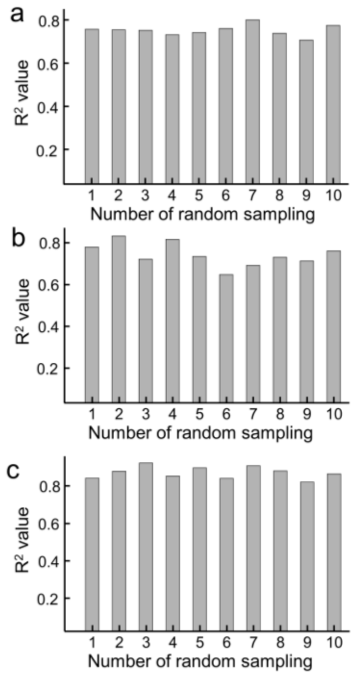

Figure S26. Evaluation of machine-learned quantification through 10 times repeated random sampling cross-validation for (**a**) uric acid, (**b**) lactate, and (**c**) tyrosine.

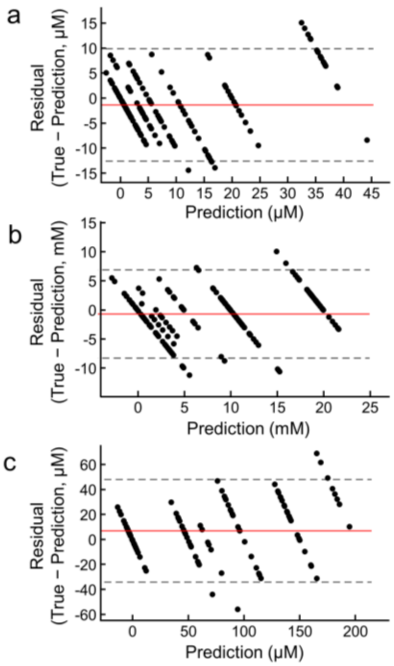

Figure S27. Bland-Altman plot for metabolic quantification model of (**a**) uric acid, (**b**) lactate, and (**c**) tyrosine. The red solid line indicates the mean bias between the actual and predicted values, while the dashed lines represent the 95% limits of agreement (mean ± 1.96 standard deviation).

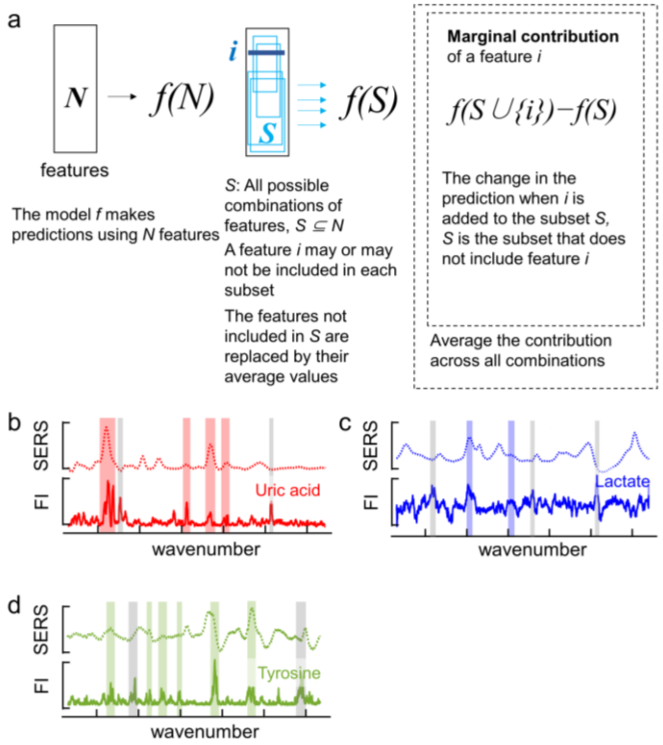

Figure S28. (**a**) Schematic illustration of SHAP feature importance calculation. Extracted SHAP feature importance (FI, solid line) and SERS spectra at 10 mM (dotted line) of (**b**) uric acid, (**c**) lactate, and (**d**) tyrosine. The bars indicate the matched SERS peaks (colored) and notches (gray).

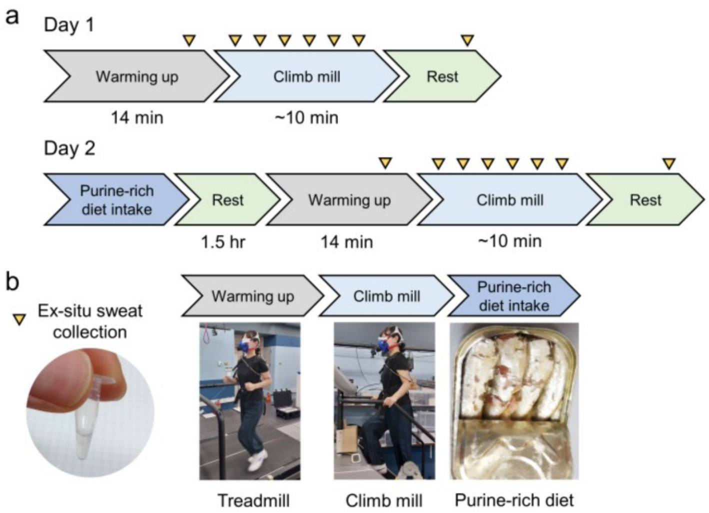

Figure S29. (**a**) Experimental protocols of the on-body evaluation. (**b**) Optical images of sweat collection using microtube (left) and assorted activities (right). The triangular markers represent the sweat collection during exercise for sensor validation.

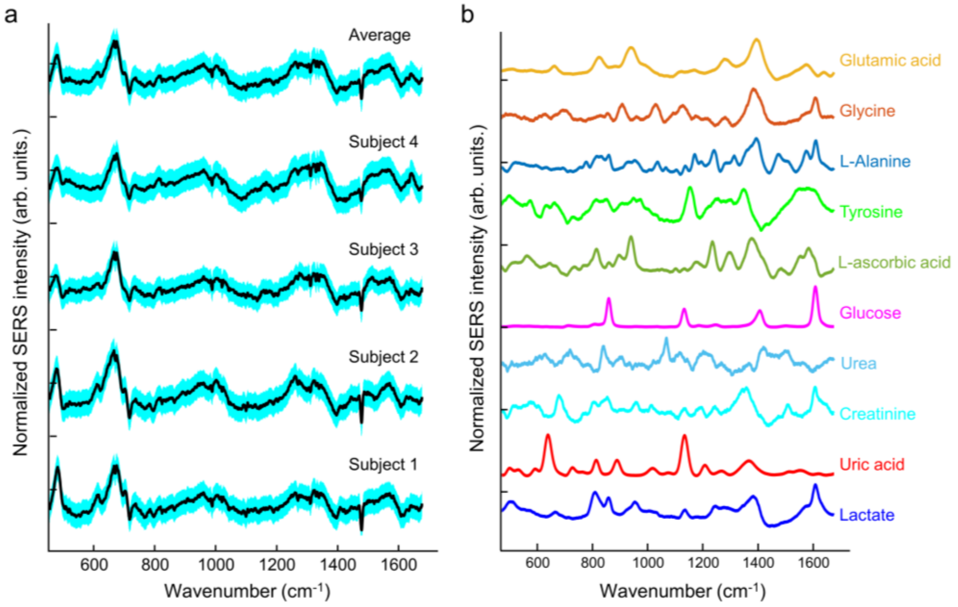

Figure S30. SERS spectra of (**a**) human sweat depending on participants and (**b**) various metabolites in sweat.

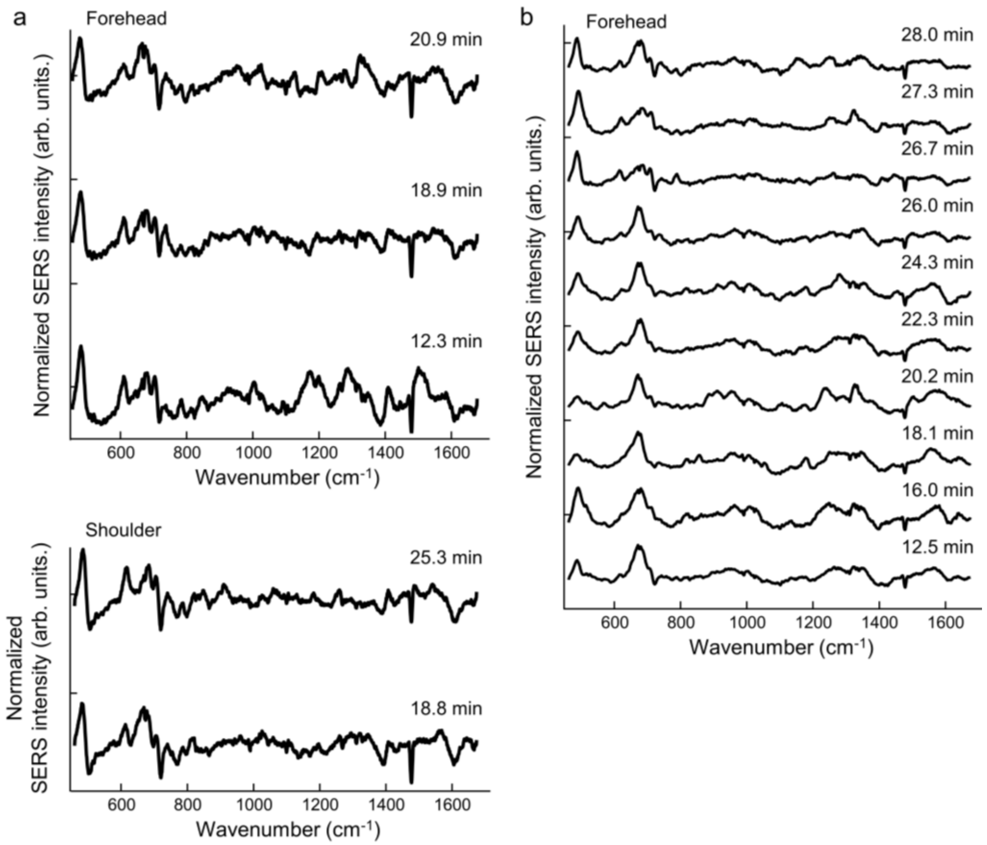

Figure S31. SERS spectra of chrono-sampled sweat under (**a**) fasting condition and (**b**) purine-rich diet intake (Participant 1). Each SERS spectrum is an average of 7 different measurements.

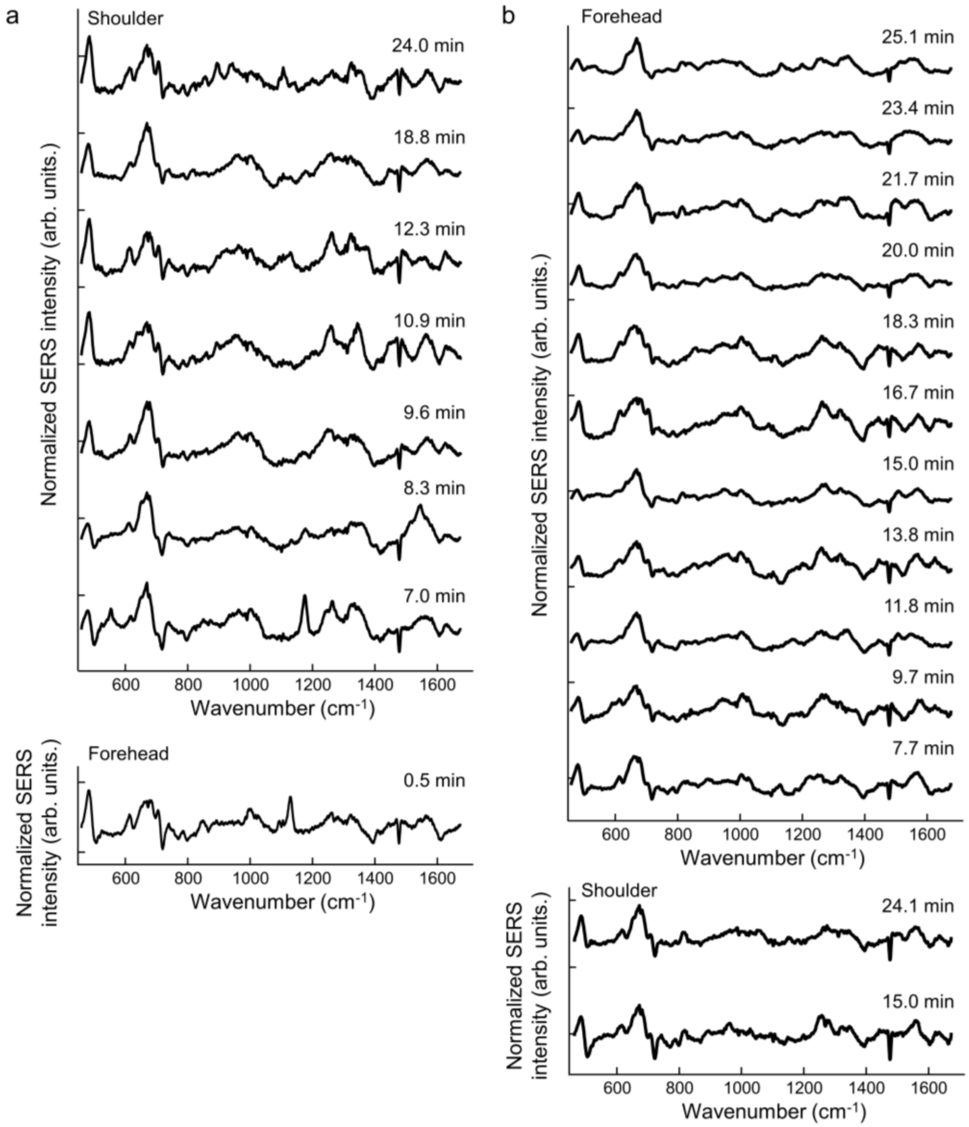

Figure S32. SERS spectra of chrono-sampled sweat under (**a**) fasting condition and (**b**) purine-rich diet intake (Participant 2). Each SERS spectrum is an average of 7 different measurements.

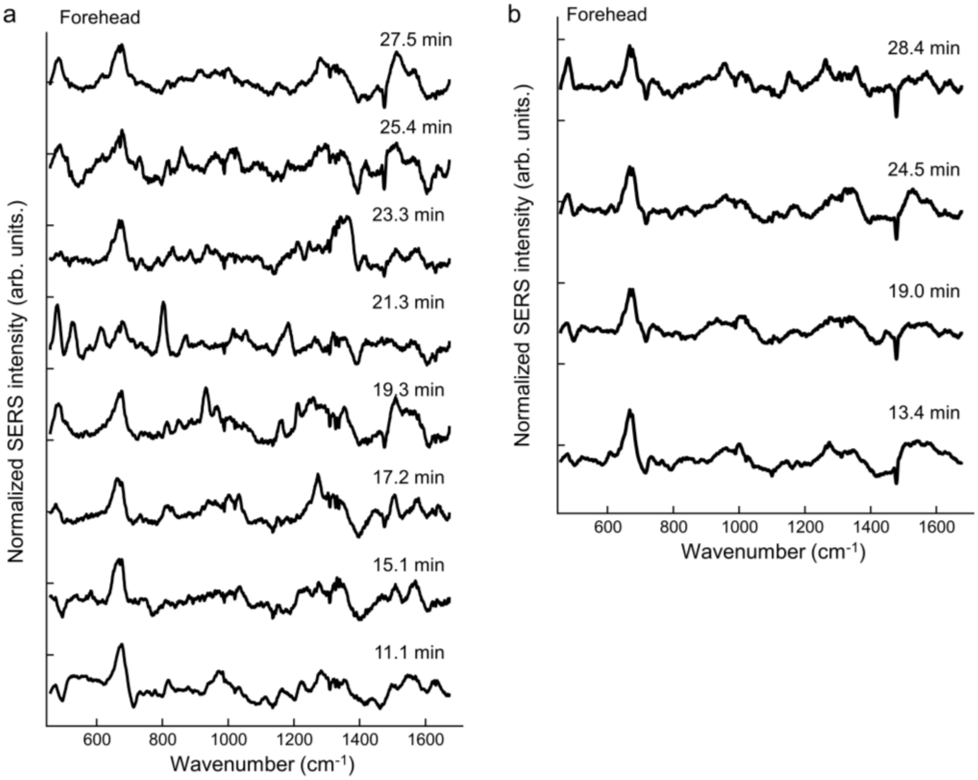

Figure S33. SERS spectra of chrono-sampled sweat under (**a**) fasting condition and (**b**) purine-rich diet intake (Participant 3). Each SERS spectrum is an average of 7 different measurements.

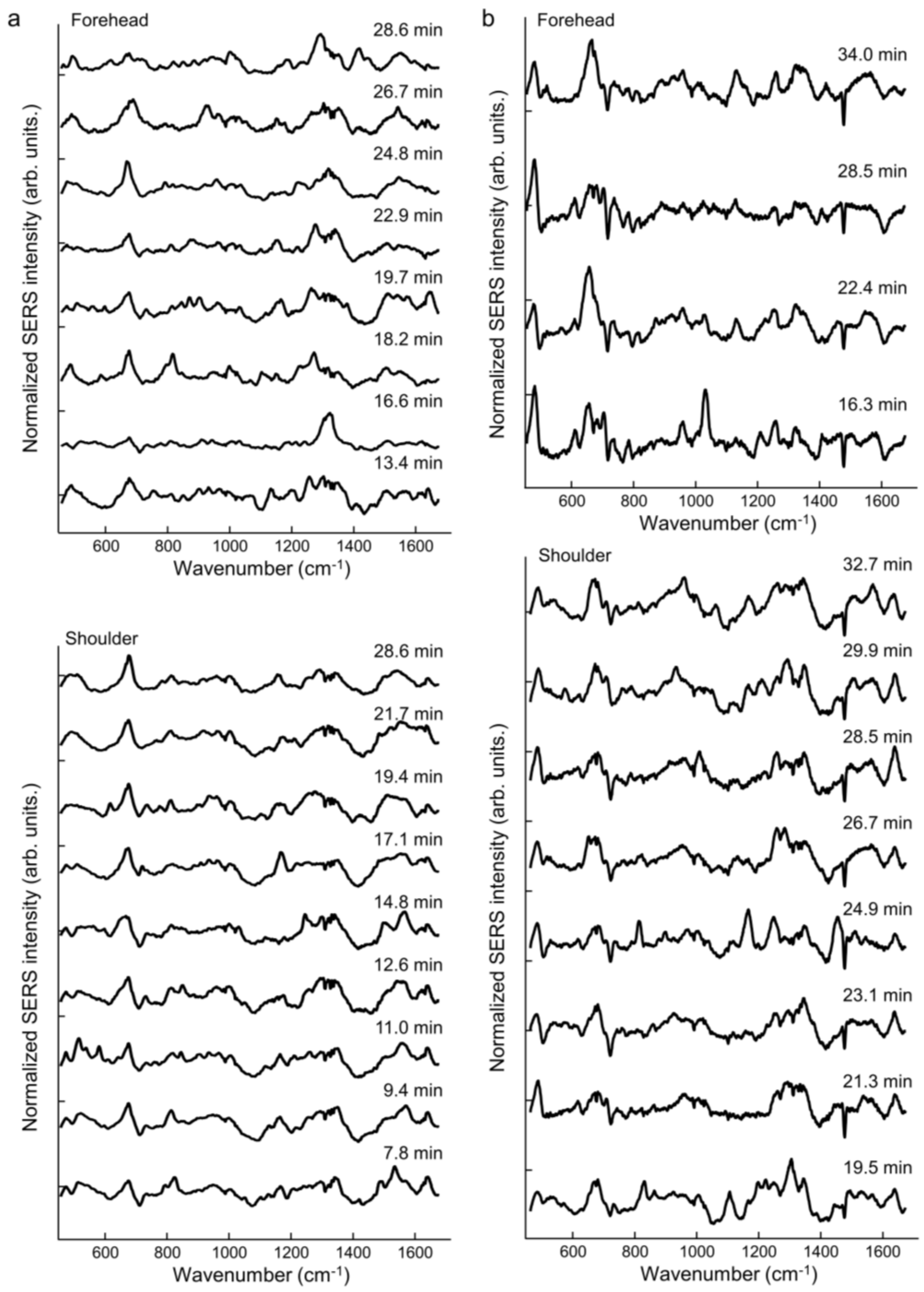

Figure S34. SERS spectra of chrono-sampled sweat under (**a**) fasting condition and (**b**) purine-rich diet intake (Participant 4). Each SERS spectrum is an average of 7 different measurements.

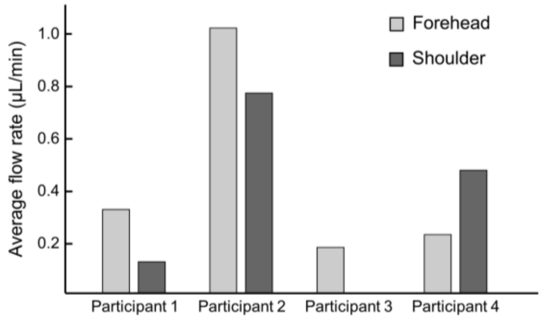

Figure S35. Measured sweat rate depends on the participants and body parts.

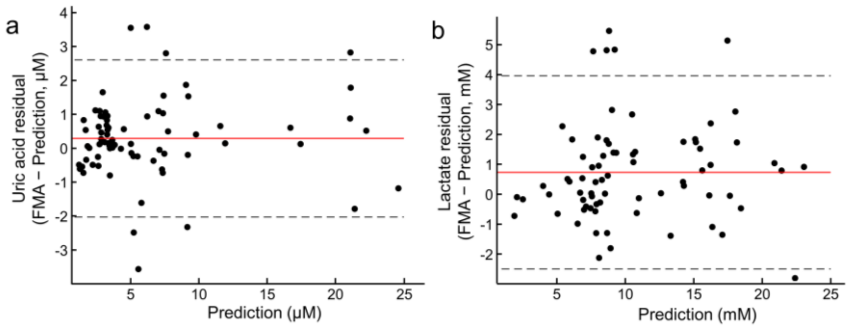

Figure S36. Bland-Altman plot for (**a**) uric acid and (**b**) lactate prediction in human sweat. The red solid line indicates the mean bias between the actual and predicted values, while the dashed lines represent the 95% limits of agreement (mean ± 1.96 standard deviation).

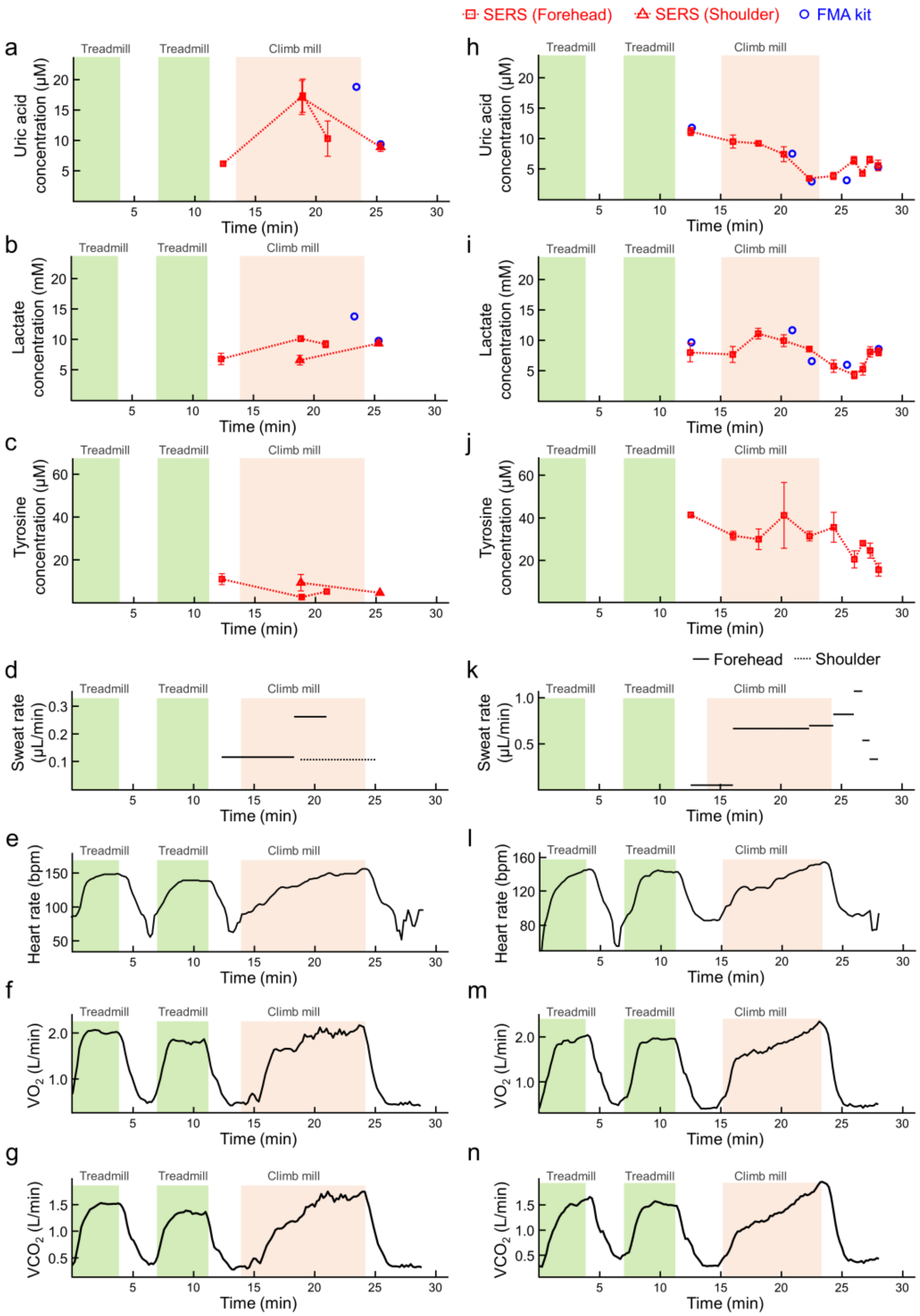

Figure S37. Chronological profiling of lactate, uric acid, tyrosine, sweat rate, heart rate, oxygen (VO_2), and carbon dioxide (VCO_2) uptake under (**a-f**) fasting condition and (**g-l**) purine-rich diet intake (Participant 1). The error bars represent one standard deviation from the mean (n=6 different SERS measurement at identical chamber).

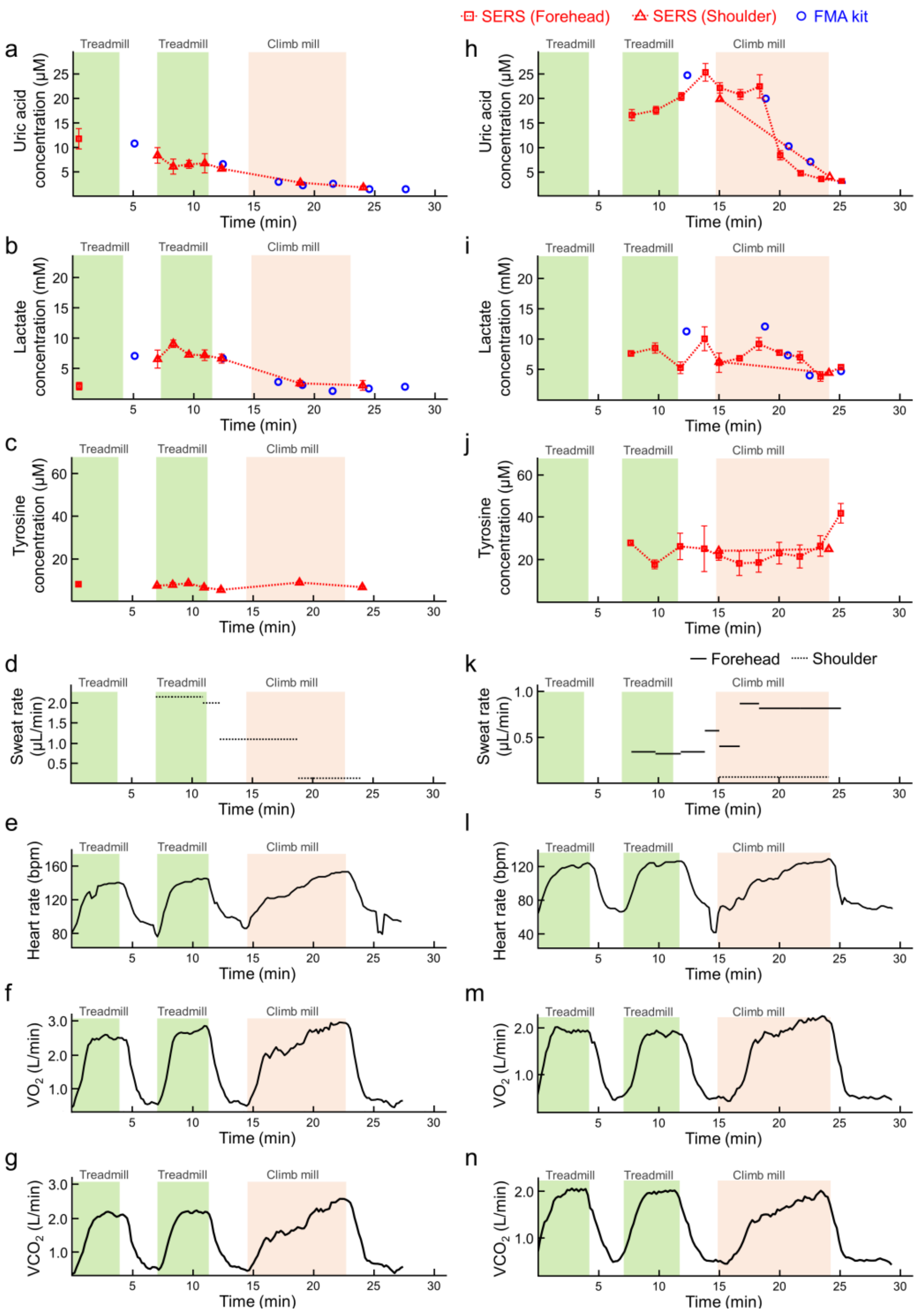

Figure S38. Chronological profiling of lactate, uric acid, tyrosine, sweat rate, heart rate, oxygen (VO_2), and carbon dioxide (VCO_2) uptake under (**a**-**f**) fasting condition and (**g**-**l**) purine-rich diet intake (Participant 2). The error bars represent one standard deviation from the mean (n=6 different SERS measurement at identical chamber).

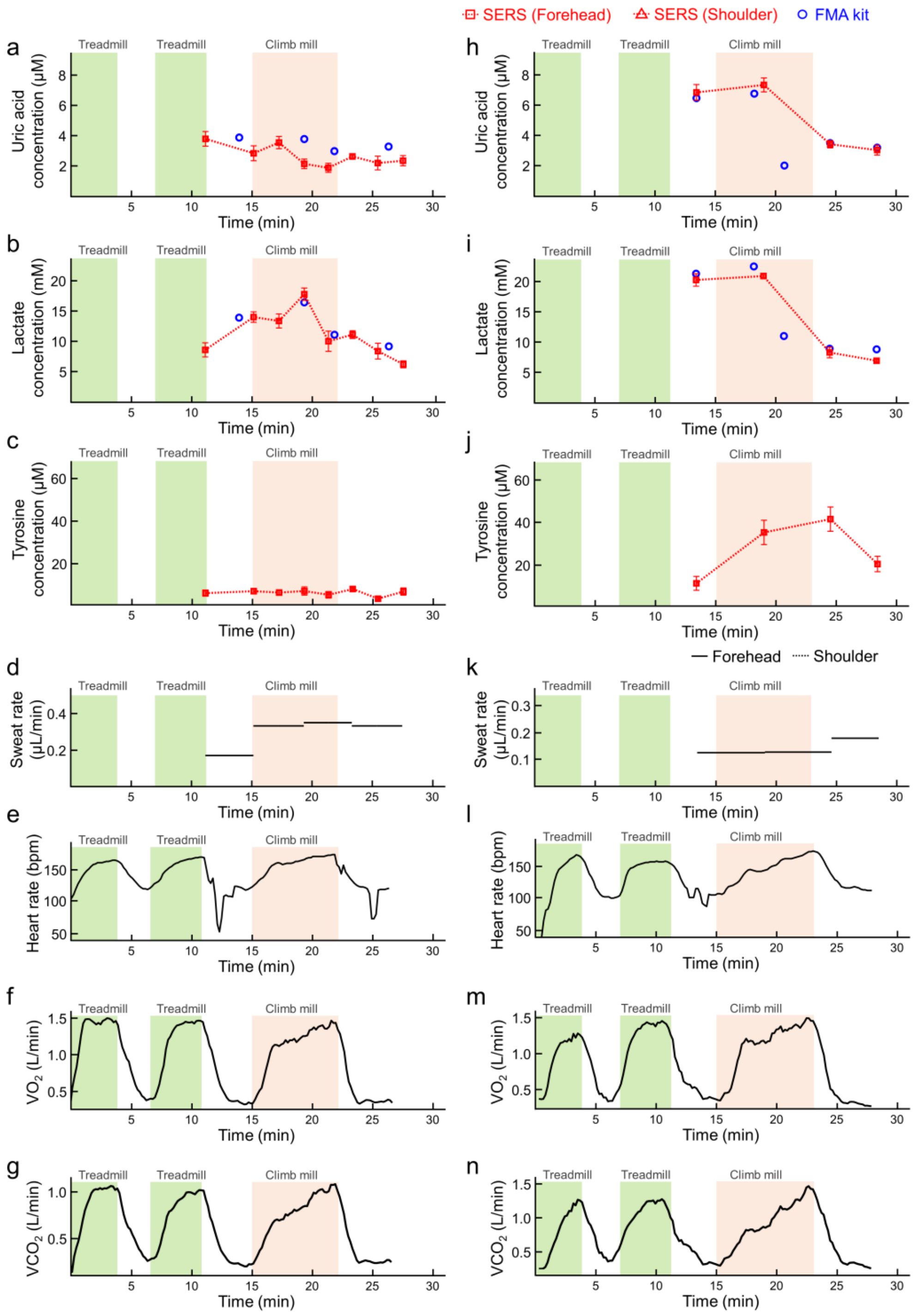

Figure S39. Chronological profiling of lactate, uric acid, tyrosine, sweat rate, heart rate, oxygen (VO_2), and carbon dioxide (VCO_2) uptake under (**a-f**) fasting condition and (**g-l**) purine-rich diet intake (Participant 3). The error bars represent one standard deviation from the mean (n=6 different SERS measurement at identical chamber).

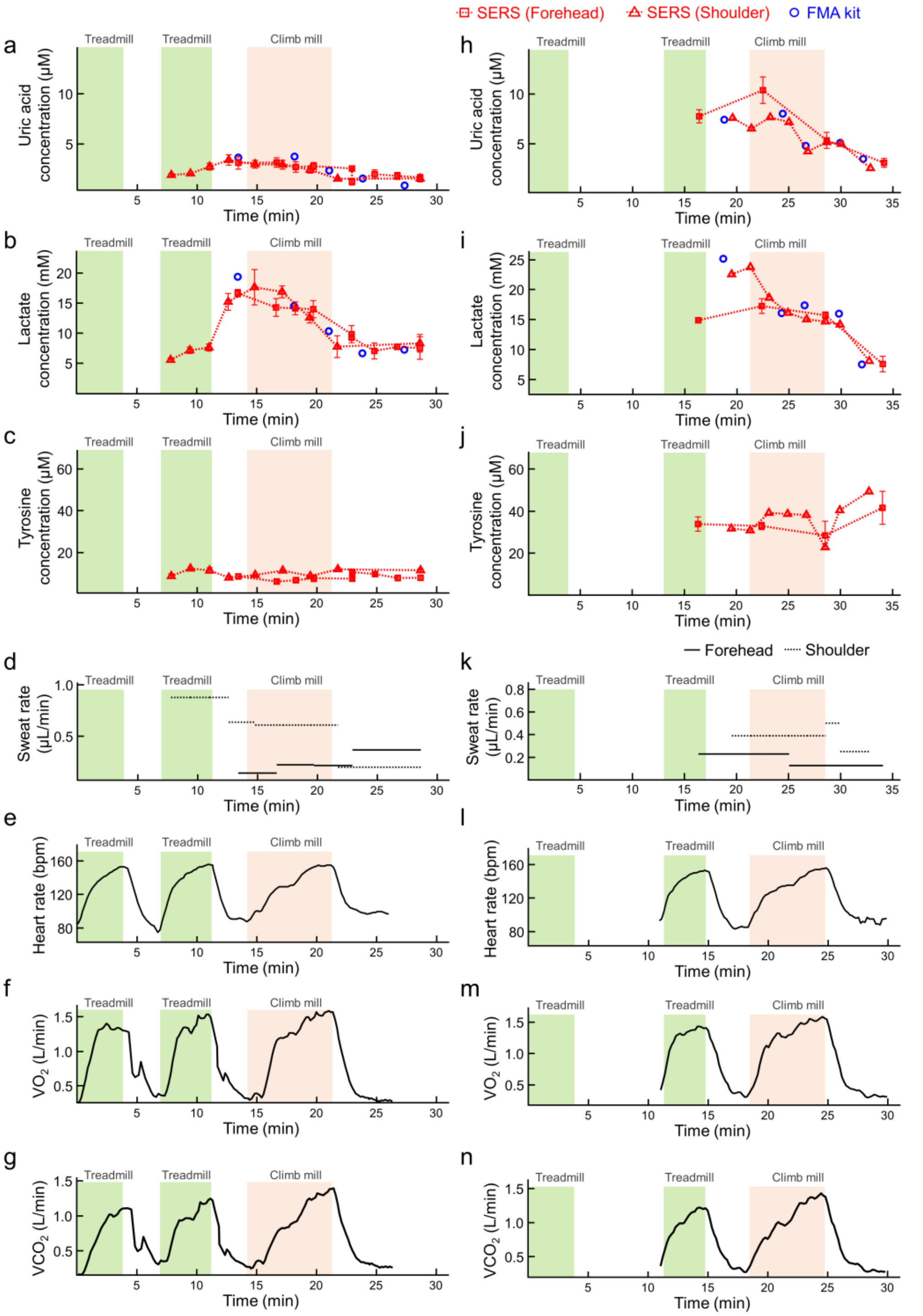

Figure S40. Chronological profiling of lactate, uric acid, tyrosine, sweat rate, heart rate, oxygen (VO_2), and carbon dioxide (VCO_2) uptake under (**a**-**f**) fasting condition and (**g**-**l**) purine-rich diet intake (Participant 4). The error bars represent one standard deviation from the mean (n=6 different SERS measurement at identical chamber).

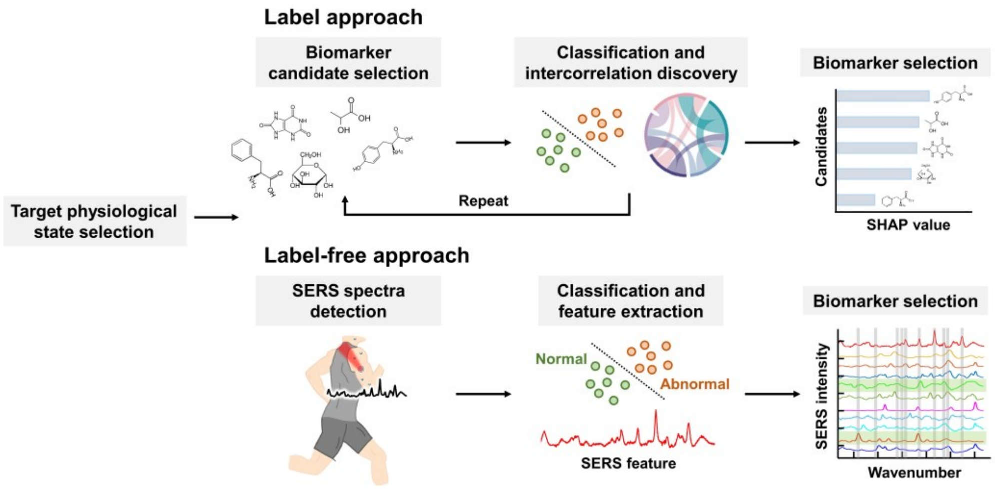

Figure S41. Schematic illustration of biomarker discovery through label-free SERS analysis

	SERS substrate	Flexibility	Functional microfluidic compatibility	Label	Analytes
CEP-SERS patch	Ag nanoislands on fluorocarbon-coated PDMS	High	High	Label-free	Multiple metabolites<br>- Uric acid<br>- Lactate<br>- Tyrosine
Y. Wang et al.,<br>*Sci. Adv.* (2021) [46]	Ag nanocube<br>on hydrogel film	High	Not demonstrated<br>(Low)	Label-free	Single drug<br>- Nicotine
U. Mogera et al.,<br>*Sci. Adv.* (2022) [58]	Au nanorod<br>on paper	High	Low	Label-free	Single metabolite<br>- Uric acid
X. He et al.,<br>*npj Flex. Elect.*<br>(2022) [54]	Ag nanomushroom<br>on Si wafer	Mediocre	Mediocre<br>(Simple assembly)	Label-free<br>& probe<br>molecule	Dual metabolites & pH<br>- Urea<br>- Lactate
J. Xiao et al.,<br>*ACS Sens.* (2023)<br>[53]	Au nanomushroom<br>on Si wafer	Mediocre	Mediocre<br>(Simple assembly)	Label-free	Single drug<br>- Acetaminophen
W. Yu et al.,<br>*Biosens. & Bioelect.*<br>(2024) [48]	Core-shell Au nanorod<br>on textile	High	Not demonstrated<br>(Low)	SERS tag<br>(Label)	Dual metabolites<br>- Lactate<br>- Glucose
Z. Chen et al.,<br>*Lab Chip* (2024) [52]	Ag nanowire<br>on paper	High	Low	Label-free	Single metabolite<br>- Uric acid
H. Ye et al.,<br>*Nano Letters* (2024)<br>[53]	Au nanoparticle<br>on PDMS	High	Mediocre<br>(Simple assembly)	Label-free<br>& probe<br>molecule	Single metabolite & pH<br>- Lactate
M. Hu et al.,<br>*Biosens. & Bioelect*<br>(2024) [55]	Au @ Ag nanocubes<br>on silk	High	Mediocre<br>(Simple assembly)	Label-free	Dual metabolites<br>- Creatinine<br>- Uric acid
H. Zhang et al.,<br>*Nano Letters* (2024)<br>[57]	Template printed<br>Au nanowire<br>on PDMS	High	Mediocre<br>(Simple assembly)	Probe<br>molecule	pH
G. Li et al.,<br>*Nano Letters* (2024)<br>[60]	Au & Al_2O_3 trimers<br>in hydrogel	High	Mediocre<br>(Simple assembly)	Label-free	Dual metabolites<br>- Lactate<br>- Uric acid

Table S1. Comparison of recent advances in wearable SERS patch for human sweat analysis. Previously reported wearable SERS patches still encounter several major challenges to accomplish precise sweat profiling: (1) Absence of an all-flexible SERS patch compatible with sophisticated functional microfluidic channels limits precise fluid control, restricting accurate quantification and time-resolved analysis. (2) The high molecular complexity of sweat hinders the multiplexed quantification of metabolites.
