# Supplementary material for: All-flexible chronoepifluidic nanoplasmonic patch for label-free metabolite profiling in sweat
Source: Nat Commun. 2025 Aug 27;16:8017. doi: 10.1038/s41467-025-63510-2 (PMC12391418; doi:10.1038/s41467-025-63510-2)
Supplement: Supplementary file 2 — Description of Additional Supplementary Files [file 41467_2025_63510_MOESM2_ESM.pdf]

Description of Additional Supplementary Files

Supplementary Data 1: SERS data for training machine-learned quantification model

Supplementary Movie1: Chrono-sample collection through microfluidic sequential sampler
