# Supplementary material for: All-flexible chronoepifluidic nanoplasmonic patch for label-free metabolite profiling in sweat
Source: Nat Commun. 2025 Aug 27;16:8017. doi: 10.1038/s41467-025-63510-2 (PMC12391418; doi:10.1038/s41467-025-63510-2)
Supplement: Supplementary file 5 — Reporting Summary [file 41467_2025_63510_MOESM5_ESM.pdf]

nature portfolio

Corresponding author(s): Ki-Hun Jeong

Last updated by author(s): Jul 15, 2025

Reporting Summary

Nature Portfolio wishes to improve the reproducibility of the work that we publish. This form provides structure for consistency and transparency in reporting. For further information on Nature Portfolio policies, see our Editorial Policies and the Editorial Policy Checklist.

Statistics

For all statistical analyses, confirm that the following items are present in the figure legend, table legend, main text, or Methods section.

n/a	Confirmed	
☐	☒	The exact sample size (n) for each experimental group/condition, given as a discrete number and unit of measurement
☐	☒	A statement on whether measurements were taken from distinct samples or whether the same sample was measured repeatedly
☒	☐	The statistical test(s) used AND whether they are one- or two-sided<br>*Only common tests should be described solely by name; describe more complex techniques in the Methods section.*
☒	☐	A description of all covariates tested
☒	☐	A description of any assumptions or corrections, such as tests of normality and adjustment for multiple comparisons
☐	☒	A full description of the statistical parameters including central tendency (e.g. means) or other basic estimates (e.g. regression coefficient) AND variation (e.g. standard deviation) or associated estimates of uncertainty (e.g. confidence intervals)
☒	☐	For null hypothesis testing, the test statistic (e.g. F, t, r) with confidence intervals, effect sizes, degrees of freedom and P value noted<br>*Give P values as exact values whenever suitable.*
☒	☐	For Bayesian analysis, information on the choice of priors and Markov chain Monte Carlo settings
☒	☐	For hierarchical and complex designs, identification of the appropriate level for tests and full reporting of outcomes
☒	☐	Estimates of effect sizes (e.g. Cohen's d, Pearson's r), indicating how they were calculated

Our web collection on statistics for biologists contains articles on many of the points above.

Software and code

Policy information about availability of computer code

Data collection	Lumerical FDTD Solutions was used for E-field calculation. Comsol Multiphysics (ver. 6.1) was used for finite element analysis.
Data analysis	Matlab R2020b was used for SERS data analysis and visualizations. The custom codes for machine-learned quantification used in this study are publicly available on GitHub at https://github.com/errory230/Linear-scaling-autoencoder. Machine-learned quantification models were tested on an A100 GPU server using Python 3.7.9 and PyTorch 1.11.0 or above.

For manuscripts utilizing custom algorithms or software that are central to the research but not yet described in published literature, software must be made available to editors and reviewers. We strongly encourage code deposition in a community repository (e.g. GitHub). See the Nature Portfolio guidelines for submitting code & software for further information.

Data

Policy information about availability of data

All manuscripts must include a data availability statement. This statement should provide the following information, where applicable:

- Accession codes, unique identifiers, or web links for publicly available datasets
- A description of any restrictions on data availability
- For clinical datasets or third party data, please ensure that the statement adheres to our policy

The authors declare that the main data supporting the findings of this study are available within the article and its Supplementary Information. In particular, the SERS spectra used for model training are provided as Supplementary Data, and the SERS spectra from human sweat samples are available as Source Data.

Research involving human participants, their data, or biological material

Policy information about studies with human participants or human data. See also policy information about sex, gender (identity/presentation), and sexual orientation and race, ethnicity and racism.

Reporting on sex and gender	Sex was recorded for all participants (2 male, 2 female). Gender was not separately assessed. As the outcomes are not sex- or gender-dependent, no sex- or gender-based analyses were performed.
Reporting on race, ethnicity, or other socially relevant groupings	Race, ethnicity, and other socially constructed groupings were not assessed, as they were not relevant to the study objectives or analysis.
Population characteristics	All participants were healthy adults aged 22 to 25 years, with no reported medical conditions or prior diagnoses relevant to the study.
Recruitment	Participants were recruited through voluntary enrollment. All participants gave written informed consent prior to participation.
Ethics oversight	This study was approved by the KAIST Institutional Review Board (IRB) under protocol number KH2024-085.

Note that full information on the approval of the study protocol must also be provided in the manuscript.

Field-specific reporting

Please select the one below that is the best fit for your research. If you are not sure, read the appropriate sections before making your selection.

☒ Life sciences ☐ Behavioural & social sciences ☐ Ecological, evolutionary & environmental sciences

For a reference copy of the document with all sections, see nature.com/documents/nr-reporting-summary-flat.pdf

Life sciences study design

All studies must disclose on these points even when the disclosure is negative.

Sample size	For on body evaluation of the CEP SERS patch, 4 healthy subjects were recruited. Sample sizes were chosen on the basis of literature standards for proof-of-concept experiments.
Data exclusions	No data exclusion.
Replication	Sweat profiling was reproducible across four participants under fasting and postprandial conditions, with consistent results in all repeated on-body tests.
Randomization	The device was fabricated using an identical process and tested under uniform conditions across all participants; thus, randomization was not applicable to this study
Blinding	Blinding was not applicable, since it had no impact on the sampling results.

Reporting for specific materials, systems and methods

We require information from authors about some types of materials, experimental systems and methods used in many studies. Here, indicate whether each material, system or method listed is relevant to your study. If you are not sure if a list item applies to your research, read the appropriate section before selecting a response.

Materials & experimental systems

n/a	Involved in the study	
☒	☐	Antibodies
☒	☐	Eukaryotic cell lines
☒	☐	Palaeontology and archaeology
☒	☐	Animals and other organisms
☒	☐	Clinical data
☒	☐	Dual use research of concern
☒	☐	Plants

Methods

n/a	Involved in the study	
☒	☐	ChIP-seq
☒	☐	Flow cytometry
☒	☐	MRI-based neuroimaging

Plants

Seed stocks	*Report on the source of all seed stocks or other plant material used. If applicable, state the seed stock centre and catalogue number. If plant specimens were collected from the field, describe the collection location, date and sampling procedures.*
Novel plant genotypes	*Describe the methods by which all novel plant genotypes were produced. This includes those generated by transgenic approaches, gene editing, chemical/radiation-based mutagenesis and hybridization. For transgenic lines, describe the transformation method, the number of independent lines analyzed and the generation upon which experiments were performed. For gene-edited lines, describe the editor used, the endogenous sequence targeted for editing, the targeting guide RNA sequence (if applicable) and how the editor was applied.*
Authentication	*Describe any authentication procedures for each seed stock used or novel genotype generated. Describe any experiments used to assess the effect of a mutation and, where applicable, how potential secondary effects (e.g. second site T-DNA insertions, mosiacism, off-target gene editing) were examined.*
